# Intra-articular antibiotic delivery combined with one-stage revision for periprosthetic joint infection: a systematic review

**DOI:** 10.1186/s42836-026-00440-4

**Published:** 2026-10-15

**Authors:** Xinyue Chang, Michelle Hilda Luk, Amy Cheung, Lawrence Chun Man Lau, Thomas Ka Chun Leung, Henry Fu

**Affiliations:** 1 Department of Orthopaedics and Traumatology, The University of Hong Kong, Hong Kong SAR, China; 2 Department of Orthopaedics and Traumatology, Queen Mary Hospital, Hong Kong SAR, China

**Keywords:** Periprosthetic joint infection, One-stage revision, Intra-articular antibiotic, Revision arthroplasty, Local antibiotic therapy, Systematic review

## Abstract

**Background:**

Periprosthetic joint infection (PJI) is a serious complication after joint arthroplasty requiring surgical and antibiotic management. One-stage revision has gained attention because it reduces the number of procedures while yielding acceptable outcomes. Intra-articular (IA) antibiotic delivery has been investigated to enhance local antimicrobial exposure and address biofilm-related challenges. However, a focused systematic review of IA antibiotic delivery combined with one-stage revision for PJI is lacking. This systematic review evaluated the techniques, safety, and outcomes of this strategy.

**Methods:**

A literature search was conducted up to 30 November 2025 across PubMed (including MEDLINE), Web of Science, Embase, and the Cochrane Library. Studies enrolling adults with PJI treated by one-stage revision combined with IA antibiotic delivery and reporting clinical outcomes with at least 12 months of follow-up were included. Study characteristics, treatment protocols, outcomes, and safety data were extracted and descriptively synthesized.

**Results:**

Ten studies were identified. IA antibiotic delivery combined with one-stage revision showed satisfactory infection-control outcomes across individual studies. Antibiotics were selected according to microbiological findings and administered via catheter infusion or direct intra-articular injection for 14–42 days. Reported complications were descriptively summarized and included catheter-related, local, surgical, and systemic adverse events. Renal-related adverse events were reported, with no documented long-term renal impairment in the included studies. Heterogeneity in treatment protocols and study designs was noted across studies.

**Conclusion:**

Intra-articular antibiotic delivery combined with one-stage revision for PJI was associated with satisfactory reported infection-control outcomes and safety findings. While the current evidence is largely retrospective and heterogeneous, further prospective comparative studies with standardized protocols would help clarify its clinical role.

**Supplementary Information:**

The online version contains supplementary material available at https://doi.org/10.1186/s42836-026-00440-4.

## Background

Prosthetic joint infection (PJI) is one of the most serious complications of joint replacement surgery, and it is the most common cause of joint revision surgery [1, 2]. PJI is highly disabling, often impairing rehabilitation, requiring prolonged antibiotic therapy and hospitalization, and carrying risks of antibiotic resistance and adverse reactions [3]. This not only adversely affects patient outcomes but also increases the burden on healthcare systems.

One-stage revision is one of the surgical techniques for treating PJI [4, 5]. Its advantages include reducing patient morbidity and scar formation associated with subsequent surgeries, shortening overall treatment time, accelerating postoperative recovery, reducing hospital stay, and lowering healthcare costs [5–8]. Recent evidence suggests that one-stage revision can achieve satisfactory infection control for chronic PJI, with comparable infection eradication to two-stage revision [9, 10]. In a recent meta-analysis, Xie et al. reported a lower reinfection rate after one-stage than after two-stage revision (10.02% vs. 14.75%) [11]. Despite surgical intervention, antimicrobial agents remain critical in treatment [12]. Local antibiotic delivery enables high drug concentrations at the infection site, potentially enhancing antimicrobial activity and biofilm penetration while reducing concerns regarding systemic toxicity associated with intravenous antibiotic therapy [13, 14]. Intra-articular (IA) antibiotic delivery, as a type of local antibiotic delivery, was first reported in 1991 as a treatment option for PJI [15]. It has been investigated across several surgical strategies for PJI. Recent clinical studies have reported satisfactory infection-control outcomes following one-stage revision combined with IA antibiotic delivery [13, 16–18].

However, current evidence regarding this combined strategy remains limited and heterogeneous. Previous reviews have generally evaluated IA antibiotic delivery across mixed revision strategies rather than specifically examining its use with one-stage revision surgery. As the effectiveness of IA antibiotic delivery may depend on surgical context, a surgery-specific synthesis focusing on IA antibiotic delivery combined with one-stage revision is necessary. Therefore, this review focuses on intra-articular (IA) antibiotic delivery with one-stage revision for treating PJI. We summarize the available evidence on clinical outcomes, safety and complications, delivery techniques, and patient characteristics. By systematically integrating current findings, we aim to provide a reference for clinical decision-making and future research.

## Materials and methods

### Study design

This systematic review was conducted in accordance with the Preferred Reporting Items for Systematic Reviews and Meta-Analyses (PRISMA) guidelines. The review protocol was registered in the PROSPERO International Prospective Register of Systematic Reviews (Registration No. CRD420251196879).

### Search strategy and study selection

A comprehensive literature search was conducted across PubMed (including MEDLINE), Web of Science, Embase, and the Cochrane Library up to 30 November 2025. No publication date restrictions were applied. Only full-text English-language studies were included. The following keywords were employed in the search: (PJI OR periprosthetic joint infection OR prosthetic joint infection OR infected arthroplasty) AND (one-stage revision OR single-stage revision OR one stage exchange) AND (antibiotic) AND (Intra-articular OR Intraarticular OR Local infusion OR Irrigation OR Negative-Pressure OR Infusion OR Catheter). Detailed search strategies for each database are provided in Supplementary Table 1 (Additional file 1). Additionally, researchers manually screened the reference lists of relevant literature to identify potentially eligible articles. This process did not uncover any additional qualifying studies. Following the removal of duplicate records, two reviewers independently screened the articles and conducted a full-text assessment of potentially eligible studies. Research identification details were not blinded to the two reviewers. Any disagreement was resolved through discussion, with a third reviewer providing a final decision when necessary.

### Inclusion and exclusion criteria

Studies were eligible for inclusion if they met the following criteria: (1) original clinical research enrolling adults (≥ 18 years) with PJI; (2) evaluation of a one-stage revision strategy combined with adjunct intra-articular antibiotic delivery as reported by the original study, with extractable one-stage data when multiple surgical strategies were reported; (3) reporting of clinical effectiveness and/or safety outcomes; and (4) a minimum follow-up of 12 months for PJI-related outcome assessment. Studies were excluded if they were non-clinical or non-original publications, lacked relevant clinical outcomes, or did not meet the predefined eligibility criteria.

### Definitions

Definitions of prosthetic joint infection were based on the diagnostic criteria reported by each individual study. Studies using consensus criteria, such as the Musculoskeletal Infection Society (MSIS) criteria [19], or explicitly stated diagnostic definitions were considered eligible. The intervention of interest was one-stage revision combined with intra-articular antibiotic delivery, including catheter-based infusion or intra-articular injection. Clinical outcomes were extracted according to the definitions reported by the original studies. Infection-control outcomes and subsequent clinical management after the index procedure, including reoperations and non-operative management when reported, were extracted and summarized based on the criteria provided by each individual study. Safety outcomes included local complications, surgical complications, and systemic complications associated with the combined treatment strategy. For infection-control outcomes, the final PJI-related follow-up assessment reported by each study was used; studies were eligible only if the follow-up duration for PJI outcome assessment was at least 12 months.

### Data extraction

Data were extracted using a standardized data extraction form. Study characteristics, including study design, sample size, baseline characteristics, and follow-up duration, were collected. Data related to intra-articular antibiotic delivery combined with one-stage revision were extracted, including delivery methods, antibiotic agents, and treatment duration. Potential overlap among studies from the same institutions or author groups was assessed by comparing study periods, patient populations, and treatment protocols. Potentially overlapping studies were retained and synthesized at the study level. Such studies were included when they provided clinically relevant information from potentially distinct cohorts or addressed different aspects of the intervention. Potential cohort overlap was recorded and considered when synthesizing the extracted data. Clinical outcomes and safety outcomes were extracted according to the definitions reported by the original studies. Microbiological and treatment-related variables were collected as background information. For studies reporting multiple surgical strategies, one-stage revision subgroup data were extracted whenever available. If subgroup-specific data were unavailable, outcomes were summarized at the reported cohort level, and subgroup-specific information was recorded as not reported (NR). Other unavailable information was also recorded as NR.

### Quality assessment

The risk of bias and methodological quality of eligible studies were assessed using the National Institutes of Health (NIH) quality assessment tools [20]. Appropriate evaluation scales were selected according to study design, with overall quality graded as good, fair, or poor. No studies were excluded solely based on methodological quality assessment. Quality assessments were conducted independently by two researchers, with disagreements resolved through discussion.

### Statistical analysis

Study characteristics and extracted data were summarized descriptively in tables. Study-level clinical outcomes, safety outcomes, and intra-articular antibiotic delivery protocols were presented descriptively according to the data reported in the original studies. Because of inconsistent reporting, clinical and methodological heterogeneity, and potential overlap between patient cohorts, no quantitative pooling or formal meta-analysis was performed.

## Results

### Study selection

**Fig. 1:**
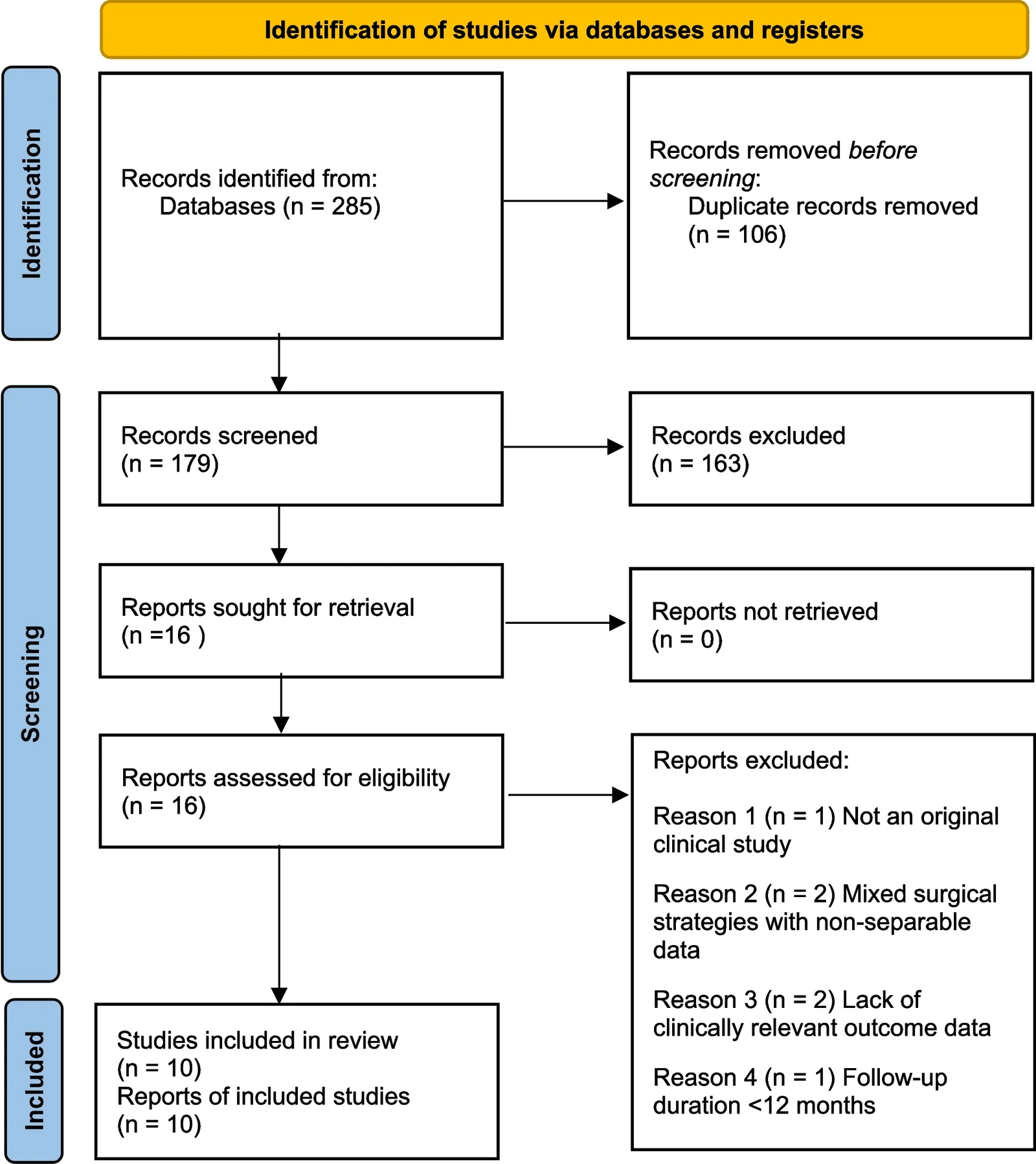
PRISMA flow diagram. n, sample size

A total of 285 records were identified through database searching. Following duplicate removal and screening, 16 full-text articles were assessed for eligibility, of which 10 studies were included in the final analysis. The study selection process is summarized in the PRISMA flow diagram (Fig. 1).

### Study characteristics

The included studies were published between 2011 and 2025 and comprised eight retrospective case series and two retrospective comparative studies involving hip and knee arthroplasty populations. All included studies were retrospective in design, with only two studies including comparative control groups [13, 21]. Follow-up duration for PJI-related outcomes was at least 12 months across all included studies. All studies evaluated one-stage revision combined with intra-articular antibiotic delivery for the management of periprosthetic joint infection. Substantial heterogeneity was observed in patient populations, study designs, and intra-articular antibiotic delivery protocols. According to the NIH quality assessment tool, eight studies were rated as fair quality and two studies were rated as good quality. Overall study characteristics and quality assessments are summarized in Table 1.

**Table 1:** Study characteristics and quality assessments

Author (Year)	Study design	Total cohort (N)	IAA + one-stage cohort (n)	Joint type	Mean FU (months)	Quality (NIH)
Ji, B, 2020 [13]	Retrospective comparative study	243	99	Hip, knee	CN 53.2; CP 59.3	Good
Li, Y, 2022 [21]	Retrospective comparative study	126	126	Hip, knee	Poly 41; Mono 39	Good
Whiteside, L, 2011 [22]	Retrospective case series	18	18	Knee	62	Fair
Ji, B, 2017 [23]	Retrospective case series	11	7	Hip, Knee	60*	Fair
Whiteside, L, 2017 [18]	Retrospective case series	30	21	Hip	63	Fair
Ji, B, 2022 [16]	Retrospective case series	78	78	Hip, Knee	85	Fair
Whiteside, L, 2012 [24]	Retrospective case series	18	10	Knee	73.2*	Fair
Vandeputte, F.J., 2025 [17]	Retrospective case series	18	18	Hip	64.2	Fair
Li, Y, 2023 [25]	Retrospective case series	32	32	Hip, knee	55.1	Fair
Ji, B, 2019 [26]	Retrospective case series	111	50	Hip	58	Fair

*IAA* intra-articular antibiotic, *N* total number of patients included in the original study, *n* number of patients in the IAA + one-stage cohort included in the review, *FU* follow-up, *Follow-up duration was reported for the overall cohort when subgroup-specific follow-up was unavailable, *Mono* monomicrobial infection, *Poly* polymicrobial infection, *CN* culture-negative, *CP* culture-positive, *NIH* National Institutes of Health

### Intra-articular antibiotic delivery protocols

All included studies evaluated a treatment strategy comprising one-stage revision surgery combined with intra-articular antibiotic delivery. The characteristics of intra-articular antibiotic delivery protocols are summarized in Table 2. Standard one-stage revision principles were reported across studies, including removal of infected prosthetic components, extensive debridement, microbiological sampling, and irrigation to reduce bacterial burden [16, 17, 21, 22, 25, 26]. Variations were observed in implant fixation methods, soft-tissue management strategies, and local antibiotic delivery approaches among studies.

**Table 2:** Characteristics of intra-articular antibiotic delivery protocols

Author (Year)	IAA delivery format	Catheter/device use/number	Timing of IAA initiation	IAA duration (days) Mean (Range)	IAA agents and dose/regimen	Systemic antibiotic
Ji, B, 2020 [13]	Infusion (hip); injection (knee)	Three-branch catheter (hip) 1; NR (knee)	Postoperative period	CN: 16 (12–20); CP: 18 (12–35)	CN: imipenem 0.5 g + vancomycin 0.5 g; CP: pathogen-directed agents	CN: vancomycin → levofloxacin/rifampicin; CP: pathogen-directed antibiotics
Li, Y, 2022 [21]	Infusion	Three-branch catheter (hip) 1; NR (knee)	Postoperative period	Mono: 15 (13–28); Poly: 16 (14–29)	Vancomycin: 0.5 g; imipenem: 0.5 g; fluconazole: 0.2 g	Vancomycin/imipenem ± fluconazole
Whiteside, L, 2011 [22]	Infusion	Hickman catheter (2/joint)	Evening of postoperative day 1	42	Vancomycin: 500 mg/10 mL	Vancomycin
Ji, B, 2017 [23]	Injection	No catheter; 18-gauge needle	Postoperative period	18 (12–30)	Fluconazole: 30 mg; voriconazole: NR	Vancomycin + antifungal therapy
Whiteside, L, 2017 [18]	Infusion	Hickman catheter (2/joint)	Postoperative day 2	42	Vancomycin: titrated to 400–500 mg; gentamicin: titrated to 40 mg/4 mL; fluconazole: NR	Fluconazole (failure case)
Ji, B, 2022 [16]	Infusion (hip); injection (knee)	Three-branch catheter (hip) 1; NR (knee)	Postoperative day 1	18 (12–35)	Vancomycin: 0.5 g; meropenem: 0.5 g; imipenem: 1 g; voriconazole: 0.1 g	Pathogen-directed antibiotics
Whiteside, L, 2012 [24]	Infusion	Hickman catheter (2/joint)	Evening of postoperative day 1	42	Vancomycin: 500 mg; gentamicin: 80 mg	Vancomycin/gentamicin
Vandeputte, F.J., 2025 [17]	Infusion	Double-lumen Hickman catheter (2/joint)	Postoperative/inpatient period (exact timing NR)	14	Vancomycin: 1000 mg; clindamycin: 600 mg; flucloxacillin: 2000 mg	Pathogen-directed antibiotics
Li, Y, 2023 [25]	Infusion (hip); injection (knee)	Three-branch catheter (hip) 1; syringe/needle injection (knee)	After microbiological confirmation	Mono: 14 (13–17); Poly: 15 (14–19)	Imipenem/meropenem: 0.5 g; vancomycin: 0.5 g (polymicrobial infection)	Imipenem/meropenem ± vancomycin
Ji, B, 2019 [26]	Infusion/injection	NR	Postoperative period	CN: 16 (12–20); CP: 18 (12–35)	Culture-negative: vancomycin 0.5 g + imipenem 0.5 g; other regimens NR	CN: vancomycin → levofloxacin/rifampicin; pathogen-directed antibiotics

*IAA* intra-articular antibiotic, *CN* culture-negative, *CP* culture-positive, *Mono* monomicrobial infection, *Poly* polymicrobial infection, *NR* not reported

Considerable heterogeneity existed in the implementation of intra-articular antibiotic delivery, particularly regarding administration methods and catheter/device configuration. Hickman catheters and three-branch catheters were the most commonly reported devices in catheter-based protocols. Catheter-based protocols used intermittent intra-articular administration, whereas some knee protocols used direct intra-articular needle injection without an indwelling catheter [23, 25]. The initiation of intra-articular antibiotic therapy varied across studies, with most protocols beginning during the early postoperative period when reported; however, one study initiated therapy after microbiological confirmation [25]. The reported duration of intra-articular antibiotic therapy ranged from 14 to 42 days. Most studies combined intra-articular antibiotic delivery with systemic antibiotic therapy, although the specific systemic regimens varied among studies. Detailed protocol characteristics are summarized in Table 2.

### Microbiological profiles and antibiotic selection

Antibiotic selection was primarily guided by microbiological profiles. Reported infections across the included studies comprised Gram-positive, Gram-negative, polymicrobial, fungal, and culture-negative infections. Vancomycin-based regimens were frequently reported, particularly for Gram-positive organisms including staphylococcal species [13, 16–18, 21, 22, 24, 26]. Broad-spectrum antimicrobial agents, including carbapenem-based regimens, were used in studies involving Gram-negative, polymicrobial, or culture-negative infections. Antifungal agents, including fluconazole and voriconazole, were reported in studies involving fungal infections [16, 18, 21, 23, 26]. Detailed microbiological profiles and antibiotic selection are summarized in Supplementary Table 2 (Additional file 1).

### Clinical outcomes and subsequent management

Infection-control outcomes were reported across all included studies after one-stage revision combined with intra-articular antibiotic delivery. The included studies reported relatively high rates of infection control, with infection-control outcomes assessed according to the criteria reported in each individual study. Subsequent surgical interventions after index one-stage revision were reported in most studies. Reported procedures included implant-retaining debridement, debridement with insert exchange, repeat single-stage revision, spacer implantation, component removal followed by reimplantation, and salvage procedures such as above-knee amputation. Subsequent management strategies varied across studies according to the clinical circumstances, and management approaches were not uniform across studies. Non-operative management strategies were reported in several studies. Chronic suppressive antibiotic therapy was described among patients managed after treatment failure or subsequent intervention. One study reported medical management without further surgery after treatment failure [23]. Detailed outcomes and subsequent management are summarized in Table 3.

**Table 3:** Clinical outcomes after one-stage revision combined with intra-articular antibiotic delivery

Author (Year)	Infection-control outcome (n/N, %)	Reoperation after index revision	Reoperation type(s)	Non-operative management
Ji, B, 2020 [13]	CN: 46/51 (90.2%)*;	Yes	Retained-component debridement; re-revision; spacer implantation	Chronic suppressive antibiotic therapy
Li, Y, 2022 [21]	Poly: 17/19 (89.5%); Mono: 101/107 (94.4%)	Yes	Implant-retaining debridement	Chronic suppressive antibiotic therapy
Whiteside, L, 2011 [22]	17/18 (94.4%)	Yes	Repeat revision/debridement	NR
Ji, B, 2017 [23]	5/7 (71.4%)	Yes	Debridement and irrigation with insert exchange	Medical management without further surgery; chronic suppressive antibiotic therapy after debridement
Whiteside, L, 2017 [18]	20/21 (95.2%)	Yes	Re-revision/redebridement	NR
Ji, B, 2022 [16]	68/78 (87.2%)	Yes	Implant-retaining debridement; single-stage re-revision; above-knee amputation	Chronic suppressive antibiotic therapy
Whiteside, L, 2012 [24]	9/10 (90.0%)	Yes	Repeat debridement/revision with implant removal followed by reimplantation	No chronic suppressive therapy reported
Vandeputte, F.J., 2025 [17]	18/18 (100%)	NR	NR	NR
Li, Y, 2023 [25]	29/32 (90.6%)	Yes	DAIR with implant retention and/or repeat single-stage revision	Chronic suppressive antibiotic therapy
Ji, B, 2019 [26]	41/50 (82.0%)	Yes	Retained-component debridement; single-stage re-revision	Chronic suppressive antibiotic therapy

Reoperation after index revision was defined as any subsequent surgical intervention after the index procedure

*IAA* intra-articular antibiotic, *DAIR* debridement, antibiotics, and implant retention, *NR* not reported

*Outcomes for the culture-positive IAA-treated subgroup were not separately reported

### Safety outcomes and complications

A summary of reported safety outcomes and complications across the included studies is presented in Table 4. Reported complications were categorized as catheter-related complications, other local adverse events, surgical complications, and systemic complications.

**Table 4:** Safety outcomes and complications reported after one-stage revision combined with intra-articular antibiotic delivery

Author (Year)	Catheter-related complications	Other local adverse events	Surgical complications	Systemic complications
Ji, B, 2020 [13]	Reported; details NR	Local rash/burning sensation	Hip dislocation; periprosthetic fracture; radiolucent line; osteolysis	Acute kidney injury; perioperative renal insufficiency
Li, Y, 2022 [21]	NR	Local rash; chronic fistula/drainage	Radiolucent line	Antibiotic-related fever; renal function changes
Whiteside, L, 2011 [22]	Catheter loss	Local inflammatory response	NR	Elevated BUN and creatinine levels requiring dose adjustment
Ji, B, 2017 [23]	NA	NR	No explicit surgical complications reported; nonprogressive radiolucent lines < 2 mm	Acute heart failure-related perioperative death
Whiteside, L, 2017 [18]	Catheter plugging	NR	Radiographic component migration without subsequent loosening	Elevated serum vancomycin levels; transient BUN/creatinine elevations
Ji, B, 2022 [16]	Catheter leakage reported	NR	Hip dislocation; periprosthetic fracture; delayed union of femoral osteotomy; arthrofibrosis; flexion instability	Calf intermuscular venous thrombosis
Whiteside, L, 2012 [24]	Catheter loss; leakage; occlusion; traumatic avulsion	NR	NR	Elevated serum antibiotic levels and/or BUN/Cr
Vandeputte, F.J.,2025 [17]	NR	Mild transient groin pressure/tenderness	Occlusion of suction drains; seroma requiring drainage; prolonged serosanguineous wound leakage	None
Li, Y, 2023 [25]	NR	Fistula; local rash	Radiolucent line at medial tibial plateau	None
Ji, B, 2019 [26]	NR	NR	Hip dislocation; postoperative periprosthetic femoral fracture; intraoperative proximal femoral fracture; osteotomy healing problems	NR

Complications were summarized according to individual study reports

*Abbreviations: IAA* intra-articular antibiotic, *NR* not reported, *NA* not applicable

In studies using catheter-based intra-articular antibiotic delivery, catheter-related complications mainly included technical or device-related issues, such as catheter occlusion, leakage, and catheter loss. Other local adverse events included local irritation or rash, fistula or drainage-related events, and transient pressure or tenderness. Surgical complications included postoperative orthopaedic and radiographic findings, such as dislocation, periprosthetic fracture, radiolucent changes, osteotomy-related complications, arthrofibrosis, instability, and wound-related complications. Systemic complications mainly involved renal function abnormalities, acute kidney injury, antimicrobial-related adverse events, elevated serum antibiotic levels, and thrombotic events. Given the variability in complication reporting and definitions across studies, safety outcomes were synthesized descriptively without calculation of a pooled overall complication rate.

## Discussion

This systematic review provides a surgery-specific evaluation of intra-articular (IA) antibiotic delivery in the setting of one-stage revision for prosthetic joint infection (PJI). To our knowledge, this is the first systematic review specifically evaluating IA antibiotic delivery within a one-stage revision framework. Ten studies were included. Individual studies reported satisfactory infection-control outcomes following one-stage revision combined with intra-articular antibiotic delivery; however, considerable variability was observed in patient selection, treatment protocols, and outcome definitions across studies. These findings suggest that IA antibiotic delivery may serve as a potential adjunctive strategy within one-stage revision for PJI. By focusing specifically on the one-stage revision setting, this review also provides a foundation for future research on localized antibiotic strategies in PJI management.

One-stage revision has become an increasingly accepted strategy for the management of PJI, with reported infection control outcomes comparable to those of two-stage revision in selected patients [1, 10, 11]. Previous studies have demonstrated satisfactory reinfection-free survival following one-stage revision, supporting its role as a surgical option for appropriately selected cases [27]. Within the one-stage revision setting, IA antibiotic delivery has been introduced as an adjunctive strategy to enhance early postoperative local antimicrobial exposure. Biofilm formation is a key factor in the persistence of PJI, as eradication of biofilm-embedded bacteria requires antibiotic concentrations 100–1000 times the minimum inhibitory concentration (MIC) [28]. Systemic antibiotic therapy may be limited by toxicity, restricting achievable local drug levels. In contrast, direct IA antibiotic delivery can achieve higher local antibiotic concentrations than intravenous therapy [29], potentially reaching levels sufficient to target biofilm-embedded organisms [30]. Moreover, IA administration may reduce systemic exposure and may allow flexible dose adjustment or temporary discontinuation when clinically necessary [21, 22].

Regarding safety, catheter-related issues were generally limited to the insertion site or surrounding soft tissue and included occlusion, leakage, dislodgement, and local inflammatory reactions. These events were reported in individual studies and varied in their occurrence and management. Indwelling catheters may theoretically serve as a portal for microbial entry and increase the risk of secondary infection [31, 32]. Fungal infections have also been reported, particularly in immunocompromised patients or in the setting of prolonged catheter retention, although such complications appear to be uncommon [31, 33]. While concerns regarding deep infection risk remain, previous studies of intra-articular pain catheters in total knee arthroplasty have not demonstrated increased deep infection rates [34]. In the included studies, catheter-related events were managed with catheter-related interventions or supportive measures when reported. Renal-related adverse events were among the most frequently reported systemic complications. However, no significant long-term renal impairment was documented in the included studies. The potential for high local antibiotic concentrations with limited systemic exposure represents a theoretical advantage of IA delivery, although comparative safety data remain limited [13, 16, 22]. Further studies with standardized protocols and more consistent adverse event reporting are needed to better characterize the safety profile of this adjunctive strategy.

Substantial heterogeneity was observed across the included studies with respect to antibiotic selection, dosing regimens, timing and duration of administration, as well as patient selection criteria, outcome definitions, and follow-up protocols. This methodological variability limited interstudy comparability and precluded quantitative synthesis. Most included studies were retrospective, involved small sample sizes, and lacked control groups. In addition, several reports originated from the same centers across different time periods, raising the possibility of partial overlap in patient cohorts. These limitations highlight the need for high-quality prospective comparative studies to better evaluate the clinical effectiveness and safety of IA antibiotic delivery in the setting of one-stage revision for PJI. At present, the independent contribution of IA antibiotic delivery beyond standard one-stage revision for PJI remains uncertain. Future multicenter prospective studies incorporating standardized surgical techniques, clearly defined IA antibiotic protocols, and consistent outcome reporting will be essential to better define its incremental clinical value.

## Conclusions

This systematic review summarized the available evidence regarding the combined use of intra-articular antibiotic delivery and one-stage revision for PJI. Individual studies reported satisfactory infection-control outcomes and safety findings. The clinical effectiveness of this combined treatment strategy requires further evaluation. Variations in study designs and treatment protocols persist across the available literature. Further prospective comparative studies with standardized protocols and consistent outcome reporting may help better define the role of this combined treatment strategy in PJI management.

## Supplementary Information

Supplementary Material 1

## Data Availability

All data analyzed in this systematic review were extracted from previously published studies. The data supporting the conclusions of this review are included within the article and its additional file.

## Abbreviations

CN: Culture-negative
CP: Culture-positive
FU: Follow-up
IA: Intra-articular
IAA: Intra-articular antibiotic
MIC: Minimum inhibitory concentration
Mono: Monomicrobial infection
MSIS: Musculoskeletal Infection Society
NIH: National Institutes of Health
NR: Not reported
PJI: Periprosthetic joint infection
Poly: Polymicrobial infection
PRISMA: Preferred Reporting Items for Systematic Reviews and Meta-Analyses
