# Supplementary material for: Intra-articular antibiotic delivery combined with one-stage revision for periprosthetic joint infection: a systematic review
Source: Arthroplasty. 2026 Oct 15;8:79. doi: 10.1186/s42836-026-00440-4 (PMC13670723; doi:10.1186/s42836-026-00440-4)
Supplement: Supplementary file 1 [file 42836_2026_440_MOESM1_ESM.docx]

**Supplementary Table 1. Database-specific search strategies**

| Database and platform | Full search strategy | Filters |
| --- | --- | --- |
| PubMed  (including MEDLINE) | (PJI OR periprosthetic joint infection OR prosthetic joint infection OR infected arthroplasty) AND (one-stage revision OR single-stage revision OR one stage exchange) AND (antibiotic) AND (Intra-articular OR Intraarticular OR Local infusion OR Irrigation OR Negative-Pressure OR Infusion OR Catheter) | None |
| Web of Science | TS=((PJI OR periprosthetic joint infection OR prosthetic joint infection OR infected arthroplasty) AND (one-stage revision OR single-stage revision OR one stage exchange) AND (antibiotic) AND (Intra-articular OR Intraarticular OR Local infusion OR Irrigation OR Negative-Pressure OR Infusion OR Catheter)) | None |
| Embase via Ovid | 1. (PJI or periprosthetic joint infection or prosthetic joint infection or infected arthroplasty).mp. 2. (one-stage revision or single-stage revision or one stage exchange).mp. 3. antibiotic.mp. or antibiotic agent/ 4. (Intra-articular or Intraarticular or Local infusion or Irrigation or Negative-Pressure or Infusion or Catheter).mp. 5. 1 and 2 and 3 and 4 | None |
| Cochrane Library | (PJI OR periprosthetic joint infection OR prosthetic joint infection OR infected arthroplasty) AND (one-stage revision OR single-stage revision OR one stage exchange) AND (antibiotic) AND (Intra-articular OR Intraarticular OR Local infusion OR Irrigation OR Negative-Pressure OR Infusion OR Catheter) | None |

All databases were searched from inception to 30 November 2025. No language, publication-date, or other database filters were applied.

**Supplementary Table 2.** **Reported microbiological profiles and intra-articular antibiotic regimens**

| Antibiotics | Reported microbiological profile |
| --- | --- |
| Vancomycin | Gram-positive organisms, particularly staphylococcal species |
| Gentamicin | Gram-negative organisms, including mixed Gram-negative infections |
| Imipenem; Meropenem | Gram-negative organisms, polymicrobial infections, and culture-negative infections |
| Clindamycin; Flucloxacillin | Gram-positive organisms and mixed infections |
| Fluconazole; Voriconazole | Fungal infections |
| Combination regimens | Polymicrobial infection; multidrug-resistant organisms; culture-negative infection |

IAA, intra-articular antibiotic.
